# Patterns of motivations and ways of quitting smoking among Polish smokers: A questionnaire study

**DOI:** 10.1186/1471-2458-8-274

**Published:** 2008-08-04

**Authors:** Alicja Sieminska, Krzysztof Buczkowski, Ewa Jassem, Katarzyna Lewandowska, Romana Ucinska, Marta Chelminska

**Affiliations:** 1 Department of Pneumonology and Allergology, Medical University of Gdansk, ul. Debinki 7, 80-952 Gdansk, Poland; 2 Department of Family Medicine, Nicolaus Copernicus University in Torun, Collegium Medicum in Bydgoszcz, ul. M. Sklodowskiej-Curie 9, 85-094 Bydgoszcz, Poland; 3 Department of Pneumonology and Tuberculosis of the Pomeranian Center of Infectious Diseases and Tuberculosis in Gdansk, ul. Smoluchowskiego 18, 80-214, Gdansk, Poland

## Abstract

**Background:**

The majority of Polish smokers declare their will to quit smoking and many of them attempt to quit. Although morbidity and mortality from tobacco-related diseases are among the highest in the world, there is a lack of comprehensive cessation support for smokers. We aimed to investigate how Poles, including the medically ill, cope with quitting cigarettes and what their motivations to quit are.

**Methods:**

Convenience sampling was used for the purpose of the study. Individuals attending several health care units were screened for a history of quit attempts. Ex-smokers were defined as smoking previously at least one cigarette/day but who have no longer been smoking for at least one month. Attempts at quitting were defined as abstaining from cigarettes for at least one day. Data on socio-demographics, tobacco use, quitting behaviors and reasons to quit from 618 subjects (385 ex- and 233 current smokers) who fulfilled these criteria were collected with the use of a questionnaire. For the comparison of proportions, a chi-square test was used.

**Results:**

In the entire study population, 77% of smokers attempted to quit smoking on their own and a similar proportion of smokers (76%) used the cold turkey method when quitting. Current smokers were more likely than former smokers to use some form of aid (p = 0.0001), mainly nicotine replacement therapy (68%). The most important reasons for quitting smoking were: general health concern (57%), personal health problems (32%) and social reasons (32%). However, 41% of smokers prompted to quitting by personal health problems related to tobacco smoking did not see the link between the two. A small proportion of ex-smokers (3%) abstaining from cigarettes for longer than a year were not confident about their self-efficacy to sustain abstinence further.

**Conclusion:**

The majority of Polish smokers, including patients with tobacco-related diseases, attempt to quit without smoking cessation assistance, thus there is a need for a broader professional help for them. There is also a lack of general information on hazards related to tobacco and further anti-tobacco campaigns in media are needed. Finally, former smokers should be given more attention and periodic inquiries regarding the smoking habit are worthwhile.

## Background

Before Communism collapsed, Poland had the highest level of tobacco consumption in the world, which resulted in an extreme level of tobacco-related diseases [1]. The democratic transformation and national health campaigns, which have begun in Poland in the early 1990s, influenced positive changes in the lifestyle of Polish society. In consequence, morbidity and mortality from tobacco declined although they still remain among the highest in the world, especially from lung cancer [2,3]. This was directly related to a gradual and steady decrease in tobacco consumption [2,4,5]. In the general population of adult men, the rate of daily smokers decreased from 52% in 1990 to 39% in 2000, and in women from 26% to 24%, respectively, however, in the years 2002–2005 this trend has not been such pronounced, with 38% smokers among men and 25.6% among women [6]. Simultaneously, the rate of ex-smokers in the years 1990–1993 increased after one decade from 14% to 20% [5,7]. It is estimated that only one anti-smoking campaign, "Let's Quit Smoking Together", conducted every year since 1991 on the annual "No Smoking Day" in November, resulted in approximately 2.5 million people quitting smoking in the years 1991–2003 [unpublished data of the Cancer Epidemiology and Prevention Division, Cancer Center and Institute of Oncology, Warszawa, Poland]. A survey conducted for the Cancer Commission of the EU demonstrated that, in 1998, Poland had the most developed anti-smoking attitude among European countries, with willingness to quit declared by approximately 70% of smokers [8]. However, for most smokers, giving up the habit is a difficult process. The majority of smokers try to quit repeatedly, cycling through multiple periods of relapse and remission [9]. Therefore, apart from preventive actions aimed at reducing the uptake of smoking, tobacco-control efforts should focus on giving smokers professional help to quit. Unfortunately, access to professional treatment of tobacco dependence is rather limited in Poland. Despite the continuing high prevalence of smoking, there is a lack of outpatient clinics offering support for smoking cessation [10]. Another problem is the insufficient involvement of Polish physicians in the treatment of tobacco-dependent patients. To date, too great a proportion of them have no adequate knowledge on smoking cessation methods and are poorly skilled in treating smokers. In consequence, physicians try to avoid any professional action, concentrating on minimal intervention and, sporadically, supporting their patients with emotional encouragement to quit smoking [10]. This disadvantageous situation has begun to change in the past several years. Poland, following other countries [11], adopted evidence-based guidelines for the treatment of tobacco dependence [10] which underline the role of individual health professionals, as well as the role of the whole healthcare system in treating tobacco dependence. Moreover, medical practitioners have nowadays many occasions to attend post-graduate courses and training sessions on professional assistance in quitting smoking.

It appears of great importance to recognize smokers' motivation to quit. The answer to the question: "What motivates smokers to quit?" might be a useful tool in tailoring a smoking cessation strategy, both for individual patients and the general population. A growing body of literature indicates that motives to quit could be divided into health, social and financial concerns, and health concern is the most important reason to quit, followed by social considerations [12]. In addition, health concern seems to be the strongest motivator to quit regardless of age, both in adolescent and elderly smokers [13,14]. In some studies, the most common reason to stop smoking was concern about future health [15,16], in others it was the occurrence of illness [17,18].

In the face of an increasing willingness to stop smoking in the entire population of Polish smokers, the purpose of our study was to investigate how Poles, including the medically ill, cope with quitting smoking and what their motivations are.

## Methods

The convenience sample was selected from the consecutive outpatients attending the Department of Family Medicine, Nicolaus Copernicus University of Torun, Collegium Medicum in Bydgoszcz (NCUT-CMB) because of a variety of health problems, patients of two pneumonology departments (Department of Pneumonology of the Medical University of Gdansk and the Department of Pneumonology and Tuberculosis of the Pomeranian Center for Infectious Diseases and Tuberculosis in Gdansk) treated because of respiratory system diseases, including tobacco-related ones, and patients of an allergology department (Department of Allergology of the Medical University of Gdansk), who in the vast majority were subjects in good condition, undergoing immunotherapy because of *Hymenoptera venom *allergy. All four departments were screening patients for past smoking and current smoking with a history of quitting attempts during routine visits.

Smoking was defined as the constant use of at least one cigarette per day. Former smokers were defined as smoking previously at least one cigarette/day but who have no longer been smoking for at least one month. Attempts at quitting were defined as abstaining from cigarettes for at least one day with the intention to quit definitively. Six hundred forty-nine subjects who fitted inclusion criteria were enrolled to the study and further asked to complete a questionnaire designed separately for former and current smokers. The questions focused on socio-demographic data, tobacco use behaviors (number of cigarettes smoked daily, duration of smoking, duration of abstinence in former smoking), history of previous quit attempts (number of quit attempts, duration of abstinence) and way of quitting (e.g., using stop-smoking treatment/self-quitting, cold turkey approach/gradual reduction of smoking). The motivation to quit, the existence of tobacco-related health problems and patients' perception of self-efficacy in quitting were also evaluated by the means shown in Table 1.

**Table 1:** Measures of quitting behaviors, awareness of smoking related symptoms and perceived self-efficacy in quitting

A subject of evaluation	Measure
Motivation to stop smoking	A checklist of the following motives for quitting (one or more answers permitted): personal health problems (+ a multiple open-ended response to specify them), family member's illness, physician's advice, somebody else's instigation, cost, health concern, anxiety about family members' health, and other motives (+ a multiple open-ended response to define them).
Smoking cessation methods used	A question: "Which of the following smoking cessation aids were you using in any of your attempts to quit smoking? The checklist (one or more answers permitted): none, nicotine replacement therapy, psychotherapy, pharmacological treatment, bioresonance and other (+ a multiple open-ended response).
Mode of quitting	The question: "What mode of quitting were you commonly using at your quit attempts?: a/quitting abruptly, b/reduction of cigarette consumption.
Health consequences of smoking	The question: Have you ever suffered from a disease caused or aggravated by tobacco? ("yes"/"no").
Perceived self-efficacy in quitting	The question: "Do you consider you have quit smoking definitively? ("yes"/"no"/"don't know").

The level of education was recorded as primary, vocational, high and university. Marital status was classified into two categories: unmarried (including widowed or divorced) and married. Self-reported standard of living was used as a proxy for income and was recorded as low, intermediate, high and excellent. Participants were classified by their current occupational status into three categories: 1. blue-collar workers; 2. white-collar workers; 3. others. The latter group included unemployed respondents, including students, never-working housewives and people who retired early due to their health problems.

Approval was obtained from the institutional research ethics committees at the Medical University of Gdansk and the Nicolaus Copernicus University of Torun.

### Statistical analysis

All data were entered into Microsoft Excel. Statistical analysis was performed using the software package Statistica 7.2. For the comparison of proportions, a chi-square test was used with continuity correction whenever appropriate. All reported values were two-sided and a p value of <0.05 was used to denote statistical significance.

## Results

Responses from 618 subjects were analyzed. The remaining 31 respondents (5%), who did not provide full information on their smoking and quitting behaviors or motivation to quit, were excluded from the analyses. Approximately half of the subjects (48%) included in the analysis were from the Family Physician Department of NCUT-CMB, 30% of subjects from an allergology department, and 22% of subjects from pneumonology departments. The characteristics of the study population by quitting status are shown in Table 2.

**Table 2:** Characteristic of the study population by the smoking status

Characteristic	Total		Former smokers		Current smokers	
	
	N/	%/mean	N	%/mean	N	%/mean
Total No.	618	100	385	62	233	38
Age						
<30 yrs.	-	52.0 ± 13.0	-	54.9 ± 13.0	-	47.2 ± 11.5
30–50 yrs.	29	5	14	4	15	6
51–70 yrs.	244	39	121	31	123	53
>70 yrs	291	47	201	52	90	39
Gender	54	9	49	13	5	2
Female	248	40	136	35	112	48
Male	370	60	249	65	121	52
Marital status						
Unmarried	145	23	81	21	64	27
Married	461	75	295	77	166	71
Missing data	12	2	9	2	3	1
Education level						
Primary/Vocational	233	37	115	30	118	51
High/University	380	61	267	69	113	48
Missing data	5	1	3	1	2	1
Standard of living						
Low	61	9	39	10	22	9
Intermediate	355	58	209	54	146	63
High/excellent	191	31	131	34	60	26
Missing data	11	2	6	2	5	2
Type of work						
Blue collar	300	48	154	40	146	63
White collar	257	42	190	49	67	29
Other	28	5	17	4	11	5
Missing data	33	5	24	6	9	4
Duration of smoking	-	23.2 ± 11.6	-	23.0 ± 12.2	-	23.5 ± 10.5
No. of cig/day	-	18.6 ± 8.7	-	19.7 ± 9.0	-	16.9 ± 8.0
1–10	143	23	72	19	71	30
11–19	102	17	60	16	42	18
≥ 20	371	59	253	66	118	51
Missing data	2	1	0	0	2	1
No. of pack/years	-	22.6 ± 17.0	-	24.2 ± 18.2	-	20.0 ± 14.3
< 20	287	45	166	43	121	52
20–39	231	37	145	38	86	37
40–60	73	12	53	14	20	8
> 60	23	4	19	5	4	2
Missing data	4	2	2	1	2	1

### Quitting behaviors, motivation to quit, and self-efficacy in sustaining abstinence

Former smokers needed usually 2–10 attempts (63%) before they achieved abstinence; 17% of them did not smoke for less than a year, nearly a half (49%) for 1 to 10 years, and every third person (33%) for longer than 10 years. However, a few (3%) of the long-term abstainers (over a year) were not confident about their self-efficacy in sustaining abstinence further.

As shown in Table 3, the majority of smokers attempted to quit without any aid, using a cold turkey approach. Nicotine replacement therapy (NRT) and pharmacological treatment were the most frequent smoking cessation methods, used by 20% of quitters. Current smokers were more likely than former smokers to use some form of aid, NRT being the most used (68% of subjects reporting aided quitting), followed by pharmacological treatment (32%) and bioresonance (14%). The use of some form of smoking cessation aid by current smokers did not differ in relation to either socio-economic factors or smoking behaviors (i.e. number of cigarettes smoked daily, years of smoking, or pack/years).

**Table 3:** Quitting characteristics and a pattern of motivation in former and current smokers

Characteristics	Total		Former smokers		Current smokers	
	
	N	%	N	%	N	%
Total No.	618	100	385	100	233	100
No. of quit attempts						
1	172	28	127	33	45	19
2–3	193	31	100	26	93	40
4–10	213	34	142	37	71	30
>10	29	5	11	3	18	8
Missing data	11	2	5	1	6	3
Max. duration of previous abstinence*						
<1 week	88	18	30	12	58	25
≤1 week<1 month	99	20	52	20	47	20
≤1 month<1 year	182	37	109	42	73	32
≥1 year	107	22	58	23	49	21
Missing data	15	3	9	3	6	2
Unaided/aided quitting						
Unaided	473	77	321	83	152	65
Aided ^y†^	145	23	64	17	81	35
NRT	88	14	30	8	55	24
Psychotherapy	16	3	14	4	2	1
Pharmacological treatment	37	6	11	3	26	11
Bioresonance	19	3	8	2	11	5
Other	9	1	6	2	3	1
Way of quitting						
Cold turkey	469	76	304	79	165	71
Reduction of cig.	125	20	60	16	65	28
Missing data	24	4	21	5	3	1
Reasons for quitting ^y^						
Personal health problem	195	32	142	37	53	23
Family member's illness	32	5	20	5	12	5
Physicians's instigation	74	12	49	13	25	11
Somebody else's instigation	70	11	38	10	32	14
Cost	94	15	42	11	52	22
Health concern	354	57	202	52	152	65
Concern about family members' health	97	16	61	16	36	15
Other	75	12	44	11	31	13

*Excluding 127 former smokers who had no previous quit attempts and stopped smoking at the first attempt

^y ^Multiple choice

^†^p = 0.0001 comparing current and former smokers

In Table 3, patterns of smokers' motivation to stop smoking are also shown. The category of other, less frequent reasons for quitting attempts included: being pregnant (5% of females), desire not to become addicted, discomfort after smoking or religious motives. The assessment of motivation to quit smoking according to selected socio-demographics was performed only in current smokers, because of possible time bias in former smokers, and is shown in Table 4.

**Table 4:** The frequencies of reasons to quit smoking in current smokers according to selected socio-demographics*

Characteristics	Reasons for quitting**															
	
	Personal health problem		Family member's illness		Physician's instigation		Somebody else's instigation		Cost		Health concern		Concern about family members' health		Other	
	
	N	%	N	%	N	%	N	%	N	%	N	%	N	%	N	%
Gender																
Female	25	22	4	4	16	14	19	17	28	25	71	63	16	14	22	19
Male	28	23	8	7	9	7	13	11	24	20	81	67	20	16	9	7
Education ^y^																
Primary/Vocational	33	28	7	6	10	8	10	8	25	21	72	61	13	11	13	11
High/University	19	17	5	4	14	12	21	19	26	23	78	69	23	20	18	16
Type of work^†^																
Blue collar	36	25	9	6	12	8	14	10	33	23	93	64	20	14	18	12
White collar	8	12	1	1	10	15	14	21	13	19	49	73	15	22	11	16
Other ^z^	5	45	1	9	2	18	3	27	4	36	6	54	0	0	0	0
Standard of living																
Low	6	27	2	9	2	9	2	9	4	18	11	50	3	14	3	14
Intermediate	34	23	6	4	17	12	20	14	36	25	97	66	16	11	16	11
High/Excellent	11	18	4	7	5	8	10	17	10	17	41	68	17	28	12	20
Marital status																
Married	33	20	9	5	17	10	22	13	34	20	109	66	31	19	20	12
Unmarried	19	30	3	5	8	12	10	16	16	25	41	64	5	8	11	17

*Two subjects have not defined their education status, 9 – type of work, 5 – standard of living, and 3 – marital status, and they are not included in the Table

**Multiple choice

^z ^The group "Other" included 4 students, 4 early retired subjects, and 3 never working wives

^y ^p = 0.043 and p = 0.024 comparing the rates of subjects with primary/vocational education vs. high/university education, who reported personal health problem or somebody else's instigation as a reasons for quitting, respectively

^†^p = 0.033 and p = 0.04 comparing the rates of blue collar- vs. white collar workers, who reported personal health problem or somebody else's instigation as a reasons for quitting, respectively

### Smokers' awareness of tobacco-related health problems

Among personal health problems reported by respondents, the most frequent trigger to quitting smoking were respiratory tract diseases (54%), including chronic obstructive pulmonary disease (19%). They were followed by cardiovascular disease (25%) and other disorders (21%). The majority of personal health problems (73%) were most likely tobacco-related, however only 41% of subjects indicating such problems as a reason for quitting were aware of this relation.

### Motivation and ways to quit in smokers who stopped smoking at the first attempt

One third (33%) of former smokers, both male and female (39% and 35%, respectively), quitted at the first attempt. Almost all of them (94%) were self-quitters using mostly (76%) the cold turkey approach. The majority of quitters at the first attempt (82%) were heavy smokers; on the other hand, more than a half (59%) had been smoking not longer than 20 years before quitting. Over one third of subjects reported a serious personal health problem (36%) as a reason for quitting. Most of these problems (85%) were related to or aggravated by tobacco, including coronary heart disease (33%), COPD (23%) and neoplasms (18%).

## Discussion

Firstly, some limitations of the study should be pointed out. The study sample was not representative of the whole Polish population, because respondents were non randomly recruited from patients of several health units. In this group, the rate of subjects prompted to quit smoking because of personal health problems, including tobacco-related symptoms, could be higher than in the whole population, mainly among patients of the Department of Family Medicine and two pneumonology departments. On the other hand, among patients of the Allergology Department, only a small proportion of subjects were admitted because of diseases related to or aggravated by tobacco, such as COPD, lung cancer or asthma, which could influence their decision to quit smoking. Furthermore, interpretation of answers to the questions related to events from the past requires some caution [12]. Respondents could have difficulty in recalling precisely some facts, for instance what exactly motivated their quitting attempts, or how many attempts they had undertaken. Finally, the reasons for quitting smoking listed in the questionnaires might not cover all possible reasons to quit, and some subjects motivated to quit by factors other than those listed might not include them in the open answer.

Since there is no representative data on ways of quitting and reasons to stop smoking in Poland, the present study, despite its potential limitations, is the only available estimate of the use of cessation aids. Additionally, these data highlight some motives of smokers that were important for taking the decision to quit smoking. A great part of former smokers repeatedly cycled from smoking to not smoking periods before the final success. These findings are consistent with the statement that quitting appears to be a dynamic process rather than a single event [19]. Polish smokers, as was previously reported [7], attain success in quitting on average at the seventh quitting attempt. In the present study, the majority of former smokers (63%) needed 2–10 quitting attempts to attain success in quitting.

The survey showed that "willpower" and the cold turkey method were the most common approaches to quitting in the study population, which is similar to the situation reported in other countries [9,20-22]. One explanation for such high prevalence of self-quitting might be that smokers do not perceive smoking cessation assistance as effective [23]. On the other hand, some studies have shown that the long-term success rate is about 5%, when smokers try to quit on their own [9]. Professional assistance increased the success rate to 33% [24]. That may explain why smokers make numerous quitting attempts before they succeed. Another reason for such a high rate of unaided quitting in Poland might be the relatively high cost of nicotine replacement therapy, bupropion or varenicline. This seems to be an important barrier to use of these drugs even if patients believe in their efficacy. It is also likely that in Poland most clinicians underperform in helping smokers to quit for multiple reasons, including lack of sufficient knowledge on tobacco dependence treatment, time constraints, lack of financial incentives, the conviction that most smokers are unable to quit or smoking by clinicians themselves. Nevertheless, current smokers were more frequently engaged in multiple strategies to assist smoking cessation than former smokers, with nicotine replacement therapy mostly preferred. This trend indicates that nowadays in Poland, despite all the imperfections of the health care system as regards smoking cessation treatment, smokers have more possibilities to support their quitting with some formal aid and probably are more aware of the effectiveness of the available smoking cessation methods. Similarly, studies conducted in the American population showed that current smokers believed that nicotine replacement would help in a successful cessation attempt, while former smokers did not believe the efficacy of these products. [25]. As regards alternative methods of smoking cessation, not recommended by the evidence-based clinical practice guidelines [10], our study demonstrated that, in the face of the predominance of the self-quitting method, they are rather not popular among Polish smokers. However, some proportion of them use alternative methods of smoking cessation, with bioresonance as the most common.

Data from a recent review of over thirty retrospective studies on motivation to quit smoking [12] indicate that health is the most important reason for quitting smoking. The results of our study, in medically ill patients however, were consistent with these findings. Taking together all four health-related factors from the list provided in the questionnaire (personal health problems, general health concern, illness of family member and physician's advice) into one general category, health appeared to be the most common motivator to stop smoking. Among health-related reasons, general concern about health relating primarily to future health (awareness of tobacco-related hazard, desire to maintain current health) was the most frequently cited reason for quitting (57%), followed by current health problems (32%), which is consistent with the results of another recent study, in which those reasons were reported on average by 53% and 42% of subjects, respectively [12].

Our survey on motivation to quit demonstrated that many smokers are not successful in quitting before the occurrence of a real health problem. This is consistent with the findings of Eiser et al. [26], who indicated that health concern is a better predictor of quitting interest and attempts than of successful quitting. McCaul et al. [12], on the basis of reviewed reports on motivation to quit from the past five decades, suggested that health concern is a major reason for quitting attempts, but thereafter smokers may notice little in terms of health benefits, which is conducive to relapse. Most of them do not experience serious diseases due to tobacco smoking, such as cancer or chronic obstructive pulmonary disease. Thus, the lack of consideration by quitters of the distant benefits of smoking cessation might be an important cause of relapse.

The contribution of real health disorders to the decision to quit smoking was demonstrated recently in the study conducted within the National Program of Early Detection and Prevention of COPD, which was realized in Poland in the years 2000–2002 [27]. The large scale voluntary spirometry screening of the Polish population with high risk for COPD detected 23% of subjects with airflow limitation. The survey on smoking cessation in current smokers among the screened population conducted a year from spirometry testing and the physician's advice to stop smoking if necessary, demonstrated a significantly greater cessation rate in subjects with airway obstruction compared to subjects with normal spirometric parameters [28]. In the present study, 33% of former smokers who had quit smoking at the first attempt were heavy smokers for no longer than 20 years, and over one third of them were prompted to quit smoking by a serious personal health problem, mostly a tobacco-related one. In this group, the vast majority of subjects were able to quit smoking abruptly and without any aid.

Following health concern and social reasons, which included concern about family members' health, somebody else's instigation and a family member's illness, the expenses on cigarettes was the least common motive for stopping smoking. It is noteworthy that even economically disadvantaged smokers infrequently reported financial reasons for quitting smoking. This paradox may confirm the relation between poverty and tobacco smoking [29], and that many smokers continue smoking even in spite of smoking-induced deprivation [5,30].

The results of our study on motivation to quit smoking might indirectly reflect attitudes to smoking in the Polish population and the positive changes in these attitudes occurring from the middle of the 1990s. We found that every fifth current smoker reported the cost of cigarettes as a reason for quitting, probably because of the considerable growth of prices of cigarettes in the past several years. However, paradoxically, smokers with a lower standard of living were not more frequently driven to quit smoking by financial concern than smokers with a higher standard. Moreover, smokers with a lower level of education and blue-collar workers were less frequently motivated by social pressure (i.e. somebody else's instigation) than better-educated subjects and white-collar workers. This indicates that people with a lower socio-economic status might be less susceptible to the ongoing positive changes in the lifestyle of Polish society and social pressure not to smoke. We also found that smokers with a lower socio-economic status were more likely to report personal illness as a reason for quitting in comparison to subjects with a higher status. This might indirectly reflect the overall higher morbidity in this population resulting in turn from the higher prevalence of smoking among people with a lower socio-economic status [5].

In addition, the study demonstrated that respondents did not have sufficient knowledge about the harmful effects of smoking and some of them poorly understood the relation between symptoms and smoking. As was previously demonstrated in the U.S., in spite of the widespread general awareness of people that smoking can lead to adverse health consequences, they did not have even a basic understanding of the nature and severity of these consequences and many smokers failed to internalize them [31]. From a public health perspective, therefore, we face an important challenge to increase smokers' awareness of the harm caused by tobacco.

The data from a number of studies on smoking cessation demonstrated that most relapses occur early in the quitting process [32,33]. However, some relapses occur months or even years after the quitting date [34-36]. In the present study, respondents most frequently relapsed after a month up to a year of abstinence. However, nearly every fifth smoker relapsed after an abstinence of longer than a year. Moreover, the survey on self-reported self-efficacy demonstrated that 3% of long-term abstainers expected a relapse. It was previously demonstrated that abstainers who reported decreased confidence after cessation concerning their ability to maintain abstinence were more likely to relapse thereafter [37]. Thus, we also face a need for screening the population of former smokers who are not convinced about their self-efficacy to sustain abstinence and who may resume smoking after long-term abstinence. Thus, it is necessary not only to ask patients if they smoke and to encourage them to quit smoking but also to ask former smokers whether they are able to sustain abstinence. Those who are doubtful about their self-efficacy to remain abstinent need a profound analysis of their motivation for not smoking and the self-reported potential risk factors for relapse.

## Conclusion

The results of the present study may provide important guidelines for developing interventions addressed to current smokers as well as to former smokers at risk of relapse. Firstly, we found that the majority of smokers, including patients with tobacco-related diseases, attempted to quit without professional assistance. Secondly, smokers were driven to quitting attempts mainly by health-related reasons, including the occurrence of health problems. Thirdly, some smokers were not aware of the relation between their illness and tobacco smoking. Finally, a small proportion of long-term abstainers from cigarettes were not confident about their self-efficacy to sustain permanent abstinence, hence still being at risk of relapse. Therefore, we conclude that current smokers should be more strongly motivated by a physician to quit smoking before the occurrence of real illness, better informed about the many health hazards of tobacco smoking, and offered of professional help in quitting. Former smokers should be given more attention and periodic inquiries regarding the smoking habit are worthwhile. Clinicians should better understand the relapsing nature of tobacco dependence and the requirement for ongoing care.

## Competing interests

The authors declare that they have no competing interests.

## Authors' contributions

AS conceived the study, participated in its design and coordination, performed the statistical analysis and drafted the manuscript. KB, KL, RU and MC all participated in the data collection phase, helped to interpret findings and contributed to the text. EJ participated in the design, coordination and supervision of the study and helped to draft the manuscript. All authors reviewed drafts of the manuscript and approved the final version to be published.

## Pre-publication history

The pre-publication history for this paper can be accessed here:


